# Tackling a disease on a global scale, the Global Parkinson’s Genetics Program, GP2: A new generation of opportunities

**DOI:** 10.1016/j.ajhg.2025.07.014

**Published:** 2025-08-19

**Authors:** Cornelis Blauwendraat, Alastair J. Noyce, Ignacio F. Mata, Laurel A. Screven, J. Solle, Sonya B. Dumanis, Ekemini A. Riley, Maria Teresa Periñan, Njideka Okubadejo, Christine Klein, Huw R. Morris, Andrew B. Singleton, the Global Parkinson’s Genetics Program (GP2)

**Affiliations:** 1 Coalition for Aligning Science, Chevy Chase, MD, USA; 2 Global Parkinson’s Genetics Program, Chevy Chase, MD, USA; 3 Aligning Science Across Parkinson’s (ASAP), Chevy Chase, MD, USA; 4 Centre for Preventive Neurology, Wolfson Institute of Population Health, Queen Mary University of London, London, UK; 5 Genomic Medicine Institute, Lerner Research Institute, Cleveland Clinic, Cleveland, OH, USA; 6 The Michael J. Fox Foundation for Parkinson’s Research, New York, NY, USA; 7 Unidad de Trastornos del Movimiento, Servicio de Neurología, Instituto de Biomedicina de Sevilla, Hospital Universitario Virgen del Rocío/CSIC/Universidad de Sevilla, Seville, Spain; 8 Department of Medicine, Faculty of Clinical Sciences, College of Medicine, University of Lagos, Idi-Araba, Lagos State, Nigeria; 9 Institute of Neurogenetics, University of Luebeck and Schleswig-Holstein University Hospital, Campus Luebeck, Luebeck, Germany; 10 Department of Clinical and Movement Neuroscience and UCL Movement Disorders Centre, UCL Queen Square Institute of Neurology, London, UK

**Keywords:** Parkinson disease, global genetics, global consortium, precision medicine

## Abstract

The need for more diversity in research is a widely recognized problem, especially in the genetics and genomics fields. While resolving this problem seems straightforward by recruiting and sequencing research participants from underrepresented populations, implementing an effort like this is complex operationally. Key considerations include ensuring equity, building capacity, and creating a sustainable research collective that works collaboratively to address local and global questions in research. Here, we provide a roadmap detailing how the Global Parkinson’s Genetics Program (GP2) is tackling the lack of diversity in Parkinson disease (PD) genetics research and also reflect on 5 years of progress. GP2 aims to be a global hub facilitating subject recruitment, sample collection, data generation, harmonization, and sharing. It also acts as a centralized target discovery hub for PD genetics worldwide. The underlying tenets of GP2 center on transparency, the democratization of data and discovery, training and career support, providing (or generating) actionable results, and creating a functional collective of PD researchers worldwide. GP2 is working with 275 research groups worldwide. There are data and samples from 265,000 subjects currently committed to the program as of May 2025. We discuss the lessons learned in this process and highlight what we view as the emerging opportunities that the program will aim to target over the next period.

## Teaser

The Global Parkinson’s Genetics Program (GP2) is an international effort to understand the genetic basis of Parkinson disease across diverse populations. GP2 aims to generate and share genetic data for over 250,000 participants globally, build research capacity worldwide, accelerate discoveries, and ensure that PD research benefits all populations.

## What is Parkinson disease?

Parkinson disease (PD) refers to a clinical diagnosis with features that correspond to recognizable neuropathological changes at postmortem (i.e., Lewy body pathology and nigrostriatal degeneration). In the modern era, this clinicopathological correlation was operationalized in the Queen Square Brain Bank (QSBB) diagnostic criteria.^1^ In 2015, an International Parkinson and Movement Disorder Society (MDS) task force proposed a two-stage approach for PD diagnosis: first, recognizing the clinical syndrome of parkinsonism (based on bradykinesia and rigidity or tremor) and second, determining the probability of PD as the cause, based on the presence of supportive criteria, the absence of exclusion criteria, and minimal red flags.^2^^,^^3^^,^^4^^,^^5^ In clinical practice, several supportive criteria for a PD diagnosis, such as an excellent response to levodopa replacement therapy and levodopa-induced dyskinesia, require follow-up of individuals. Despite the agreed clinical basis for the diagnosis, PD has highly heterogeneous clinical manifestations and progression.

In the first few years following diagnosis, most individuals respond well to symptomatic treatment and can continue their normal activities. However, PD progresses, and many affected individuals will develop autonomic and neuropsychiatric symptoms, loss of postural stability, and falls. Many individuals, particularly the elderly, require support at home and admission to care facilities. A recent composite analysis of PD progression based on a clinic referral series has shown that 8 years from diagnosis, around 8% of individuals with PD have died, 25% have developed significant problems with their balance, and 25% have developed cognitive impairment or dementia.^6^^,^^7^ The follow-up of older population-representative incident Parkinson cohorts shows a cumulative rate of dementia, loss to follow-up, and death due to dementia of 47%, with a median follow-up of 7 years and a mean age at diagnosis of 69 years.^8^

The focus of basic science research in PD has been on disease cause and risk, understanding the basic risk factors that lead to familial and sporadic disease. This has led to tremendous insights into disease biology, with the definition of alpha-synuclein (α-syn), lysosomal, and mitochondrial-based disease pathogenesis. However, the focus of therapeutic trial research has been in preventing disease progression—after diagnosis—and alleviation of symptoms. Less is known about the biology of progression. The Braak hypothesis of PD, which states that sporadic PD is initiated through an infection introduced through the nasal cavity and first affects the peripheral nervous system before progressing to the central nervous system,^9^ has been influential in that cross-sectional pathological data have been assembled into different stages from the brainstem to the cortex, with progressively more extensive pathology suggesting that in life, PD may progress through clinical disease stages involving more severe and more widely distributed pathology^10^ (for a more complete discussion, see Carceles-Cordon et al.^11^). However, although PD dementia correlates with advanced Braak Lewy body stages, there is a shortage of carefully characterized individuals with PD with postmortem data, which would allow validation of the Braak hypothesis; further, studies that incorporate the considerable effects of copathologies will be important.^12^^,^^13^^,^^14^ This notwithstanding, the Braak hypothesis has been interpreted as relating to the spread of pathology rather than to differential neuronal susceptibility, and the concept of pathological spread has been supported by biochemical, cellular, and animal model-based assays, confirming that synthetic and endogenous abnormal α-syn can trigger pathology in previously unaffected cells and tissues.

Pathologic, genetic, and, most recently, biomarker data support a diversity of underlying causes for PD or Parkinson syndrome. Large cohort studies from both the QSBB and the Banner Brain Bank indicate that a substantial proportion of individuals clinically diagnosed with PD have underlying tau-predominant progressive supranuclear palsy (MIM: 601104) pathology.^15^ Similarly, individuals with *PRKN* (MIM: 602544), *ATXN2* (MIM: 601517), *ATXN3* (MIM: 607047), and *MAPT* (frontal-temporal dementia [MIM: 157140]) mutations have been reported to have levodopa-responsive clinical PD. Yet, pathogenic variants in these genes are not usually associated with Lewy body pathology. These neuropathological findings have been corroborated by the development of the α-syn seed amplification assay (α-syn-SAA).^16^^,^^17^^,^^18^ In recent work, approximately 5%–15% of individuals clinically diagnosed with PD are α-syn-SAA negative, suggesting that they do not have Lewy body disease (MIM: 127750).^19^^,^^20^ As we will discuss later, if divergent pathology reflects divergent pathobiological mechanism(s), this pathological diversity will reduce the power of both large-scale mechanistic (genome-wide association studies [GWASs]) and clinical trial/therapeutic studies, perhaps most immediately where the drug target is α-syn. Notably, there is a relationship between genetics and pathology; an increased rate of α-syn-SAA-negative individuals carrying primary pathogenic *LRRK2* (MIM: 609007) variants (it is estimated that 22%–33% of individuals with PD with *LRRK2* mutations are α-syn-SAA negative^19^^,^^21^). This initial finding is likely the first of many *in vivo* advances that will define the disease by its underlying biology. This definition may not immediately be helpful in the clinical care and management of PD; however, it is a fundamentally important step in understanding the varied mechanistic bases of PD and in the development and application of mechanistically based therapeutics (precision or stratified medicine).

The definition of PD is in a state of flux and may appreciably differ between the clinical and research world; this is primarily due to rapid advances in the availability of biomarker anchors for pathological change, which avail a biological, rather than clinical, definition of disease. Abnormal aggregation characteristics of α-syn in cerebrospinal fluid (CSF) and skin are the basis of α-syn-aggregation assays, which appear to differentiate individuals with PD from healthy individuals and non-synuclein-based PD mimics. However, 5%–10% of the healthy unaffected population seems to be positive for these assays as well (in an age-dependent fashion). The emergence of better biomarkers for PD has prompted proposals for new biological classifications and/or staging criteria that incorporate genetics, α-syn-focused biomarkers, and neurodegeneration on imaging, irrespective of the presence or absence of clinical features.^22^^,^^23^ This shift in the field toward earlier detection based on the presence of biomarkers reflects similar shifts in other complex diseases, both neurological (e.g., Alzheimer disease and multiple sclerosis) and non-neurological (e.g., cancer and rheumatoid arthritis).

The burden of PD appears to be increasing. Prevalence, mortality, and disability are on the rise, even after taking account of an aging global population (https://www.healthdata.org/research-analysis/gbd). Compared to Alzheimer disease, with a prevalence of 694/100,000, the prevalence of PD is 139/100,000 and, thus, considerably lower. Of note and currently ill-explained is, however, the large difference in percentage increases from 1990 to 2021, which is moderate for Alzheimer disease (3.2%) and very high (60.7%) for PD. The great rate of increase appears to be in low-middle-income countries rather than high-income countries. Possible reasons for the apparent increase in burden could be due to changes in environmental factors (e.g., pesticide, volatile solvent, and air pollution), lifestyle, and comorbid changes (e.g., smoking behavior, infectious exposure, and type 2 diabetes) or improving ascertainment of affected individuals in the developing world (e.g., better awareness, better access, and better training of healthcare workers). Most published literature supports the observation that men are more likely to be affected by PD than women^24^; however, not all studies show this excess prevalence in men, and male-female differences may be less apparent in some parts of the world.^25^ Again, regional differences in PD burden by biological sex may be real (due to sex-specific biological differences, lifestyle, and comorbidity differences) or apparent (due to differential access to care and cohort recruitment).

## General overview of PD genetics

### What has been discovered in the PD genetics field so far?

In the last 30 years, there has been considerable investment and progress in our understanding of the genetic basis of PD.^26^ More than a dozen genes have been identified that contain high-risk or disease-causing mutations, and more than 100 independent genetic variants have been identified that contain risk alleles for disease.^27^^,^^28^^,^^29^ These findings have been a major component of the foundational knowledge on which basic functional research rests. Pathogenic variants in several genes are considered the main causes (and/or high-risk factors) for PD; these include *LRRK2*, *SNCA* (MIM: 163890), *RAB32* (MIM: 612906), and *VPS35* (MIM: 601501), where one pathogenic variant is enough to substantially increase the risk for disease (autosomal dominant), and *PRKN*, *PINK1* (MIM: 608309), and *PARK7* (MIM: 606324), which only cause disease when both copies of the gene harbor pathogenic variants (autosomal recessive). It is important to note that, as previously mentioned, pathogenic variants in these genes are all associated with the clinical syndrome called PD, and interestingly, the disease linked to mutations in each of the 7 genes is clinically variable (www.mdsgene.org) and sometimes pathologically variable. Some general themes persist, however; individuals with *LRRK2* or *VPS35* pathogenic variants are more likely to be normosmic and have slower progression, a reduced chance of dementia, and variable Lewy body pathology.^30^^,^^31^ Individuals with *SNCA* pathogenic variants often have a more rapid progression and an earlier onset, almost always develop dementia, and always have Lewy body pathology.^30^ Individuals with bi-allelic pathogenic variants in *PRKN*, *PINK1*, or *PARK7* typically have early onset and slower progression, with those harboring bi-allelic *PRKN* variants not always exhibiting Lewy body pathology.^32^^,^^33^^,^^34^^,^^35^^,^^36^

In addition to these 7 genes, there are >10 genes reported to contain pathogenic variants that cause PD. In some cases, these are rare and/or cause more atypical PDs (e.g., *DNAJC6* [MIM: 608375], *SYNJ1* [MIM: 604297], *VPS13C* [MIM: 608879], *PLA2G6* [MIM: 603604], *FBXO7* [MIM: 605648], *ATP13A2* [MIM: 610513], and *POLG* [MIM: 174763]). A third category persists, where the proposed gene association with disease lacks replication or may have significant and prolonged data-refuting association, and therefore, the balance of evidence strongly suggests they do not contain PD-causing mutations (e.g., *UCHL1* [MIM: 191342], *HTRA2* [MIM: 606441], and *TMEM230* [MIM: 617019]). Recently, individuals with pathogenic variants in *PSMF1* (MIM: 617858) were reported to present with an autosomal recessive inheritance pattern with a very early onset.^37^

Progress in identifying highly penetrant mutations has been carried out through family-based studies. Two genes have also been identified that contain relatively common protein-coding variants that confer moderate (∼2–5×) risk for disease: *LRRK2* and *GBA1* (MIM: 606463). Following the identification of disease-causing variants in *LRRK2* (e.g., c.4321C>G [p.Arg1441Gly], c.4321C>T [p.Arg1441Cys], c.4322G>A [p.Arg1441His], and the relatively common c.6055G>A [p.Gly2019Ser]), sequencing studies revealed a small number of coding variants in *LRRK2*, most notably c.7153G>A (p.Gly2385Arg) and c.4883G>C (p.Arg1628Pro), that are not disease causing but rather increase the risk for disease by approximately 2-fold. Both variants were identified in several Asian populations, and both are relatively common (>2% population frequency).^38^^,^^39^

The second gene known to contain moderate risk variants for PD is *GBA1.* Coding variants in *GBA1* were implicated in PD risk due to astute clinical observation. *GBA1* mutations have been known for years to cause the autosomal recessive lysosomal storage disorder Gaucher disease (MIM: 230800).^40^ Following the observation that the parents of individuals with Gaucher disease appeared to have PD more often than would be expected, heterozygous *GBA1* variants were established as a significant risk factor for PD.^41^^,^^42^^,^^43^ There is a range of PD-risk variants in *GBA1*, some, but not all, of which are also associated with Gaucher disease; these variants confer varied risk for PD. Individuals with PD who carry a *GBA1* variant tend to have a slightly faster progression, higher risk for dementia, and heavier burden of non-motor symptoms in general and almost always have Lewy body pathology.^40^

Significant progress has been made in identifying common PD-risk genetic variants, which are typically low-risk variants. To date, more than 100 genetic risk loci have been identified.^27^^,^^28^^,^^44^^,^^45^^,^^46^ In contrast to family-based genetics, identifying these loci has rested on genome-wide genotyping in large cohorts of affected and unaffected individuals. This work rests on the common disease common variant hypothesis that an appreciable component of genetic risk for common disease comes from a large number of common variants, which individually confer small amounts of risk but cumulatively represent significant risk. Notably, within the >100 common variant risk loci identified, this work has also revealed associations at genes known to contain rare disease-causing or moderate risk variants, demonstrating a continuum of risk alleles in genes such as *SNCA*, *LRRK2*, *VPS13C*, and *GBA1*, including high-, medium-, and low-risk variants. This phenomenon, pleomorphic risk loci, illustrates a mechanistic spectrum across the range of risk alleles and that risk can be a continuum, even within variability at the same gene.^47^ Implicitly, this has profound implications for therapeutic approaches centered on genetic hits in disease. This idea is now supported by empirical evidence that anchoring therapeutic targets on a foundation of unequivocal human genetic evidence dramatically improves the likelihood of therapeutic success, regardless of the effect size of association that led to the target identification.^48^

While much progress has been made in understanding the common variant genetic architecture of PD, much remains to be done. To date, the described risk variants only represent ∼30% of the heritable component of the disease. Based on other traits of similar heritability (for example, human height), it is expected that there will be many thousands of risk variants for PD, and these will be identified with sufficiently powered studies.^29^^,^^49^^,^^50^ Further, and perhaps most importantly, the complex genetic work has been performed almost exclusively in populations of Northern European ancestry. Some notable exceptions are a multi-ancestry meta-analysis of genome-wide association and Asian and African ancestry GWASs, which identified genetic associations likely related to background variation in risk allele frequency.^27^^,^^28^^,^^44^ However, the compelling findings from these studies illustrate the importance of and opportunities for continuing to understand the genetic basis of disease in all populations.

An expansion of genetic discovery in underrepresented populations (URPs) is not only a matter of equity but also a scientific imperative. In monogenic disease, understanding mutation rates, founder events, reduced penetrance, and discovering pathogenic variants or genes associated with disease is a valuable and worthwhile endeavor. This work provides new insights into the biology of disease, highlights new populations for precision therapeutic interventions, and supports accurate and comprehensive return of results for individuals with disease. In the context of genetically complex PD, early data show clearly that there are significant differences in the genetic architecture of PD across populations.^27^^,^^28^^,^^44^^,^^51^^,^^52^ These differences provide compelling advantages in fine-mapping existing risk loci: they nominate novel risk factors, help us understand the underlying biology of known disease-linked genes, and immediately provide previously unavailable therapeutic opportunities.^27^^,^^28^ A recent illustrative example, the identification of the non-coding rs3115534^G^ risk factor for PD in African ancestry individuals and the rapid dissection of this variant revealed not only a new mechanism, protein reduction due to missplicing, for disease risk but also a new population for *GBA1-*based clinical trials.^27^^,^^53^

## How can we use the genetic associations with PD?

Given the known heritable component of PD and the current paucity of knowledge regarding the genetic basis of disease in URPs, there is much to be found in the genetics of PD. Often-posed questions include the following: to what end? What is the purpose of this genetic discovery? Many risk loci have been found where the mechanism remains unknown, and the risk loci being identified are individually of a rather small effect size; why continue to find more genetic risk loci? It is worth taking some time to consider these questions.

The purpose of genetic discovery in disease is multi-fold. There is increasing interest in the use of genetics for the prediction of disease risk (e.g., polygenic risk scoring). We know that the heritable component of PD is not 100%, so genetics alone will rarely work for disease prediction; however, including genetics as part of a multimodal predictor will be useful for both risk and mechanistic subtyping, consequent therapeutic matching, and disease prognosis.^54^^,^^55^^,^^56^

Genetic risk factors provide a window into the pathogenesis and etiology of the underlying disease biology. These findings give immediate biological insight and are the basis for much of the field’s downstream mechanistic research. As each gene/locus is identified, it provides information on the functional network that is important in disease and the directionality of effect that risk/protective alleles confer to that network. Each causal variant and risk locus provides additional insight into the complex biology of disease, such as with the identification of risk variants at *TMEM175* (MIM: 616660), which has spurred new pathophysiological investigation and insights.^57^^,^^58^^,^^59^^,^^60^ These follow-up studies highlight that variants in *TMEM175* contribute to PD by impairing lysosomal function and affecting α-syn accumulation. This has elucidated the role of TMEM175 in lysosomal potassium channel activity, and its specific genetic variants influence neuronal health and the progression of the disease, pointing toward new therapeutic targets for PD.

Thus, genetics supports the selection and success of therapeutic targets, case selection based on matching mechanisms to therapeutics, and disease prediction. These combine to support selecting the right therapy for the right patient and deploying this at the right time.

By definition, risk loci identified by GWASs are relatively common, and generally, these risk loci are individually of modest effect size. There needs to be clarity around the concept of effect size and biological relevance. It is often thought that the genes or processes targeted by these risk alleles are of proportionally modest importance in the disease process. This is a logical fallacy, albeit a common one. The size of the risk effect is specific to the variant being interrogated and does not reflect the importance of the gene or process that it affects in the disease. As noted above and illustrative of this concept, many loci are pleomorphic in risk; for example, *SNCA*, *GBA1*, *LRRK2*, and *VPS13C* each contain a variety of risk alleles that confer dramatically different risk profiles within the same gene.^29^^,^^61^^,^^62^^,^^63^^,^^64^^,^^65^^,^^66^^,^^67^ It is important to note that our knowledge of controlling cell and disease state-specific gene expression and post-translational modification is in its infancy, and multiple dispersed risk variants (and environmental factors) may converge on the same gene or pathway. Currently, GWAS annotation relies on relatively small-scale datasets for gene expression. In the case of known rare and common variants, the effects are mediated through the same gene. Thus, a gene can be central to a disease process, but the risk alleles identified by the GWAS may have a modest effect size. The allele and its magnitude of effect are unimportant unless the variant, rather than its effector gene, is the target of therapeutic intervention.

Because of the established statistical criteria for declaring a new genome-wide association locus (*p* value < 5E−8), the confidence that there exists a variant that confers risk for disease is extremely high, and this level of confidence is more important than effect size when pursuing related therapeutic opportunities. This is elegantly demonstrated by real-world empirical data that show that genetically supported therapeutic approaches are approximately two to three times as likely to succeed as those without genetic support.^68^ This work has recently been extended to show that this relationship holds, irrespective of the associated risk effect size of the underlying genetic evidence.^48^ Unequivocal genetic evidence positively predicts therapeutic development success, but the effect size associated with this genetic evidence does not.

In PD, the majority of genetic association work has been performed with risk for PD as an outcome. As detailed above, this has led to identifying more than 100 independent risk variants. Work looking at the genetic basis of other PD-related features has been modest but has included age at onset, progression, comorbid features, pathologically defined PD, and modulation of penetrance and risk in individuals with mutations in either *LRRK2* or *GBA1*.^6^^,^^7^^,^^69^^,^^70^^,^^71^^,^^72^^,^^73^ In many instances, these studies have been hampered by limited data and small sample sizes; however, several associations have been revealed. Interestingly, the variants (either individually or combined as a polygenic risk score) that confer general risk for PD are generally associated with a lower age at onset of PD^74^ and with the risk of disease in individuals with *GBA1* or *LRRK2* pathogenic variants.^69^^,^^70^ There are some marked differences in the genetic architecture of certain features of PD; for example, variability at the *MAPT* locus is strongly associated with risk for disease but does not affect age at onset. This disparity in the architecture of risk and age at onset is the exception rather than the rule and is likely indicative of an unusual mechanism of action at the *MAPT* locus; these differences are likely to be informative. Concerning progression, there is an overlap between risk and progression alleles, particularly common and rare variants at *GBA1*. Still, there are also non-PD risk variants that have been identified from progression analysis that represent targets for gene therapy, including *APOE* (MIM: 107741), *LRP1* (MIM: 107770), *RIMS2* (MIM: 606630), *TBXAS* (MIM: 274180), and *SYT11* (MIM: 608741). However, most are lacking large-scale replication.^6^^,^^7^^,^^75^ It is worth noting that in the past decades, we have learned a lot about disease risk caused by genetics; however, there is still much to discover. Taken together, these results show that genetics is a fundamentally important step in understanding the basis of and risk for disease; as such, it serves as a foundation for the development and deployment of mechanistic-based therapeutics. Because of the fundamental importance of genetics, there is a social imperative to expand genetic discovery to global populations. As evidenced by the power of mapping genetic risk across populations, there is also a scientific advantage.

## Enabling global PD genetics by creating a research collective: GP2

The Global Parkinson’s Genetics Program (GP2, https://gp2.org/) is an international research initiative aimed at understanding the genetic basis of PD, multiple system atrophy (MIM: 146500), Lewy body dementia, progressive supranuclear palsy, corticobasal syndrome, and prodromal PD (e.g., REM sleep behavior disorder and hyposmia).^76^ It is a resource program of the Aligning Science Across Parkinson’s (ASAP; https://parkinsonsroadmap.org/#) initiative, which is managed by the Coalition for Aligning Science (CAS; https://www.aligningscience.com/) and implemented by The Michael J. Fox Foundation for Parkinson’s Research (MJFF; https://www.michaeljfox.org/). Launched in 2020, GP2 has a clear mission: to expand understanding of the genetic basis of PD dramatically and to do so in a global context.

**Figure 1:**
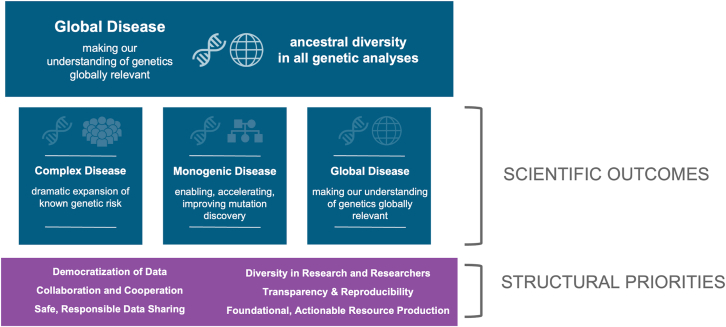
Schematic representation of scientific outcomes and structural priorities of GP2

The path to this mission centers on interwoven scientific outcomes and structural priorities (Figure 1). The former is an accelerated and dramatic expansion of our understanding of the genetic basis of PD by generating and analyzing genetic data from more than 200,000 participants to create resources and make discoveries across the spectrum of PD risk, from causative to risk variants. An imperative in achieving this aim is to do so in a globally relevant way; GP2 has prioritized accelerating discovery in groups traditionally underrepresented in PD genetics research. The structural priorities are the foundation of GP2’s operations. They serve to create equity, accelerate discovery, and create a foundation for therapeutic development that is relevant to this global disease.

GP2’s research governance is multi-layered and emphasizes collaboration and transparency. The steering committee provides overall scientific direction and oversees the program’s activities. The activities of GP2 are overseen by various working groups composed of specialists from different disciplines. These groups are responsible for specific aspects of the program, such as those focused on complex disease, monogenic disease, URPs, ensuring equity and transparency in ongoing research projects, training, data and code dissemination, operations, and compliance.

**Figure 2:**
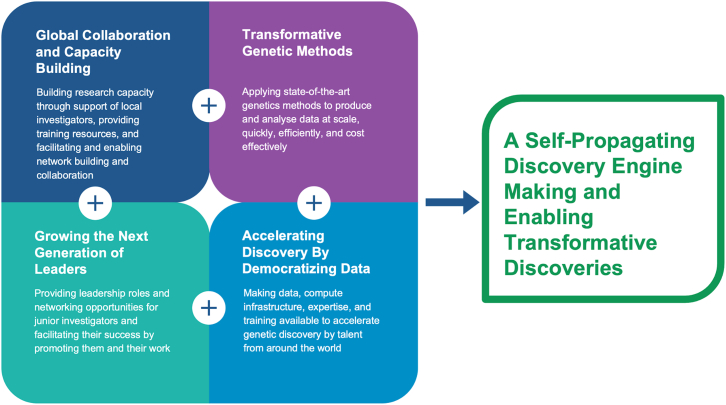
Schematic representation of four prioritized actionable areas of GP2

At its core, GP2 is a global collaborative endeavor. We believe that to accelerate research in underrepresented groups sustainably, it is essential to create an international research community. GP2 has prioritized four actionable areas to achieve this (Figure 2): global collaboration and capacity building, applying transformative genetic methods, growing the next generation of leaders, and accelerating discovery by democratizing data. Combined, these create a globally dispersed pool of leaders and researchers with the skills and resources to perform state-of-the-art genetics research. The intended net result is a self-propagating discovery engine for PD genetics.

The operational considerations for maintaining a research collective of this scale are substantial, and a great deal of work is performed by a series of working groups, including those leading issues specific to historically URPs, ethical and legal compliance, data analysis, clinical and genetic data harmonization, data and code democratization, and training and networking.

**Figure 3:**
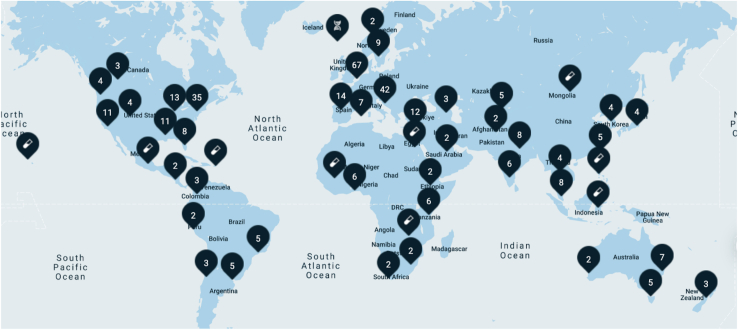
Global overview of current active locations of GP2, with over 60 actively involved countries

With >400 members from >200 sites in >60 countries worldwide (Figure 3), maintaining collaboration and communication is a challenging but essential component of GP2.^77^^,^^78^ This is done at several levels. On a day-to-day basis, GP2 has a central communication hub for members, where key information is shared, such as ongoing analytical projects and who is leading them. GP2 also hosts in-person meetings. In 2023, the Annual Investigator Meeting in Copenhagen hosted 271 researchers from 59 countries. In 2024, GP2 hosted regional investigator meetings in Cartagena (Colombia), Kuala Lumpur (Malaysia), and Casablanca (Morocco), totaling >600 attendees from >80 countries to engage with researchers from key regions of interest for building collaborations and infrastructure to support genetic research.

A particularly important component of GP2 is the training programs. These provide a network for investigators and a growing global pool of distributed talent to build new skills and capabilities at centers worldwide. The GP2 trainee network has more than 250 members. More formally, GP2 has funded 13 PhD students and 12 master’s students, each working on GP2-relevant research projects. To further build research capabilities, GP2 has created a series of training opportunities. These include >70 online courses, translated into up to 100 different languages, that are mainly, but not exclusively, focused on data analysis. Further, the GP2 training group has run data analysis workshops in Mexico, Colombia, Kyrgyzstan, Georgia, Brazil, Morocco, India, and Malaysia that use the model of “training the trainer” so that talented trainees are identified in initial workshops and taught how to continue training others.

GP2 has prioritized enabling research by the investigators who are collecting samples from individuals with PD. Part of this enabling strategy is represented by the training discussed above; however, in addition, GP2 emphasizes building local research capacity. This has taken the form of physical capacity, for example, supporting the construction of a biorepository in Lima, Peru, with a second planned for Lagos, Nigeria. It also includes local support; GP2 has provided research support, analytical support, and custom training for projects proposed by GP2 members worldwide. Lastly, it includes infrastructure. GP2 data are stored using a cloud-based platform, which enables three important components: standardization, all GP2 analysis is performed on the same infrastructure; collaboration, because it is an online platform, collaboration is accessible regardless of location; and cost, GP2 covers the computational cost of approved projects/analyses performed by GP2 members. Thus, investigators worldwide have the financial support, infrastructure, and training to perform complex genetic analyses. An effective example that demonstrates the power of this approach comes from the multi-site collaboration that led to the identification of a significant new risk factor for PD in Africa.^27^

Transparency is a core structural priority of GP2, which operates based on a no-surprises policy. Every GP2 investigator can use their GP2-generated data to ask their own research questions. If they choose to work across a broader set of data from cohorts outside of those they contributed to or seek substantial analytical support, the project must be reviewed by the Project Proposal Working Group. This group aims to ensure equity, transparency, and quality across GP2. As project proposals are submitted and reviewed, the group fundamentally asks three questions: (1) is this a reasonable use of resources (cost/benefit), (2) are the appropriate people involved (i.e., are the appropriate GP2 members involved in or aware of analysis of their data), and (3) does this incorporate a training component? The Project Proposal Working Group works iteratively with submitters to ensure these criteria are met. As projects are reviewed and approved, they are publicized in the GP2 newsletter, on the GP2 hub, and at in-person meetings. Currently, there are 60 project proposals from more than 50 investigators from 42 countries on six continents.

Additionally, GP2 is deeply committed to creating lasting societal impact by investing in sustainable local capacity, particularly in underrepresented regions. This includes building research infrastructure at participating sites; supporting independent grant applications by investigators in URP regions; fostering global collaborations, especially within the Global South; and providing targeted training in critical areas such as bioinformatics, where substantial gaps remain. These efforts are designed to foster long-term scientific growth and collaboration that will endure well beyond the lifespan of the project.

## Where is GP2 now?

GP2 is in its fifth year of operations. Clinical and genetic data from GP2 are released approximately 3 times per year. Currently, dense genotype and whole-genome sequencing data have been generated and made available from approximately 83,000 individuals in GP2 data release 10.^79^ In total, there are data and samples from 265,000 subjects committed to the program. GP2 is working with 275 sites worldwide (Figure 3) and is actively engaging with countries that are not represented on the GP2 map to continue global coverage. GP2 has encountered various legal constraints when working globally; however, solutions that allow for collaborative data-sharing solutions compliant with these diverse regulations are possible. If your country is not represented and you are interested in working with GP2, please contact us at info@gp2.org.

The first proof-of-principle results have already been published, with the original large-scale multi-ancestry GWAS for PD and the initial African ancestry-specific PD GWAS locus, including rapid translational follow-up.^27^^,^^28^^,^^53^ Several additional genetic studies will be released in 2025, including GWASs for each included ancestry and large-scale, clinically based GWASs. It is expected that in the coming years, >50 risk variants (global and ancestry specific) will be identified through GWASs, as well as several additional causal variants and genes. Notably, GP2 has also served as a robust and efficient validation and replication cohort, testing reported associations.^80^^,^^81^^,^^82^^,^^83^

## The immediate path forward for GP2 and lessons learned so far

GP2 is an ongoing learning experience on all levels (from funder to local investigators), which we consider a significant operational strength. We can try approaches, evaluate progress, correct course, and halt or accelerate components at any time. This type of flexibility is impossible without funders who have a long-term view, provide generous resources, are reliable/stable, and prioritize the result. Below, we list some of our key lessons learned.

### Stable funding is key

GP2 is a resource program of the ASAP initiative, which had funding committed for multiple years from the Sergey Brin Family Foundation through the Michael J. Fox Foundation. If it were not for the scale, flexibility, and style of this support, GP2 would not be able to operate efficiently or with the long-term and flexible vision necessary to capitalize on opportunities while maintaining stability for GP2 members. CAS and MJFF, through the ASAP initiative, are critical research partners who work with us to ensure the success of GP2. This relates to how success is measured, given that this is a new concept, and typical grant reporting strategies do not apply here. Therefore, the primary measurements of success are (1) increasing genetic discovery for PD, (2) sharing all generated data openly, (3) enabling coordinated engagement of investigators across six continents, and (4) creating opportunities for a new generation of global researchers in science and leadership.

### Balance centralized efficiency and decentralized equity

There are inevitable efficiencies and advantages in centralizing aspects of a large collaborative endeavor. These range from greater coordination and speed in operational decisions to value savings in data generation at scale. GP2 aims to balance these efficiencies with the imperative to put data and research opportunities into the hands of contributing investigators around the world. The nature of this balance has, and continues to, evolve. Much of the data production (genotyping and sequencing) is centralized through service providers, and we anticipate that this will continue because of the advantages in cost and, importantly, in standardization and harmonization. Analysis, on the other hand, becomes more decentralized as the program continues, in large part because of the evolving capabilities and skills of GP2 members worldwide. Balancing programmatic needs and efficiencies with a major tenet of the program, enabling research capability worldwide, is a theme the program addresses continually.

### Operations take time and investment

Large, international collaborations require significant operational investment. GP2 was initiated at the outset of the COVID-19 pandemic; this gave the program breathing room to develop operations, policies, and an organizational structure designed to meet the needs and philosophy of the program. While GP2 operates with a sense of urgency and a commitment to speed and efficiency, putting the initial operational construct into place took well over a year; further, an operations committee meets regularly, including in person ∼3 times a year, to discuss, recommend, and implement operational initiatives and changes. The time and effort required for this work are significant.

### Gain trust and build local infrastructure

Gaining trust can be an understandably complex process given the myriad global inequalities, particularly in URPs. Acknowledging that GP2 cannot resolve global inequality, we, directly and indirectly, support more than 80 URP investigators. For most new collections, GP2 includes an infrastructure component to support these efforts, ranging from physical resources to developing technical expertise and the training of new talent. Additionally, a major initiative undertaken in the last year has been regional investigator meetings in South America, Asia, and Africa; these meetings were aimed at engaging researchers from these communities, promoting collaboration, highlighting opportunities, and, perhaps most impactfully, listening to the needs and ideas of the individual communities. The needs raised by GP2 members have ranged broadly, including addressing the lack of appropriate scales or tools for certain regions, workshops on data analyses and variant interpretation, generation of standardized language for consent, and creation of collaborative networks through sabbaticals and training opportunities.

### Identify champions

Given that GP2 is a global endeavor, it is key to have international representation at every level of the organization. We have found that an essential component of global success has been to identify talent across all career stages and provide opportunities to lead and grow. Providing long-term training opportunities and long-term support to these individuals has been transformative for GP2 and a critical component of our success so far. Long-term support of trainees within GP2 varies based on the needs of each individual and region, including supporting key student contributors, creating master classes to enable skill building, supporting growth for non-scientific staff, providing material support for investigators to grow their networks and pursue their research, and embedding emerging and established leaders in decisional positions. GP2 supports the up-and-coming generation of researchers by pairing students with experienced leaders in the field and empowering trainees to take on additional leadership roles within GP2 (e.g., serve as junior working group leads and spearhead analyses). In addition, GP2 has funded PhD and master’s studentships and multiple scientific sabbaticals all over the world to train the next generation of genetic experts globally. It has also been important to identify established leaders who are committed to the goals and approach of GP2 and who are willing to provide time and support, often without realizing an immediate professional return.

### Remain critical and flexible

Science and scientific knowledge evolve, and challenges and opportunities emerge continuously. At its foundation, GP2’s ability to react flexibly stems from the stable, goal-oriented funding by which the program is supported. A foundational tenet of GP2, central to the initial proposal, was to be able to remain flexible and try new approaches. Inherent in this was the commitment to self-evaluate, identify failures quickly, and course correct. Once again, this is done with our partners at ASAP and MJFF and across the consortium, tracking progress, refining goals, and quickly identifying emerging opportunities and upcoming challenges. Based on member feedback, this has led us to take on new challenges, such as exploring the return of results across different regions and partnering with clinical testing initiatives such as PDGENEration.^36^

## Conclusion

PD is a complex and heterogeneous neurodegenerative disorder characterized by clinical symptoms such as bradykinesia, rigidity, and tremor, with largely predictable neuropathological changes, including Lewy body formation and nigrostriatal degeneration. There are currently no disease-modifying therapies. Advances in genetic and biomarker research have deepened our understanding of PD’s pathogenesis, revealing a wide spectrum of genetic risk factors. We believe genetic insights will help refine disease classification, facilitate earlier diagnosis, and pave the way for targeted therapeutic interventions to modify disease progression.

In order to develop targeted precision or stratified therapies, our understanding of this disease needs to match the global nature of PD; it is therefore critical that we as a community invest time and effort in understanding the basis and influences of disease in every population. This knowledge not only serves to ensure equity in treatment development and application but also provides critical insight into the disease as a whole that cannot easily be made by looking at populations in isolation.

GP2 is a global collective that aims to address the need to perform global research and leverage the opportunities provided by such an approach. GP2’s path has been complex but remarkably rewarding, and while there is a long way to go, the program holds enormous promise in generating insights, fostering collaborative and coordinated science, and giving underserved and emerging investigators the opportunities they deserve and have earned to lead the way forward.

## Acknowledgments

GP2 is funded by the 10.13039/100018231ASAP initiative and implemented by 10.13039/100000864The Michael J. Fox Foundation for Parkinson’s Research. For a complete list of GP2 members, see https://doi.org/10.5281/zenodo.15748014. A.B.S. is supported by research grants from The Michael J. Fox Foundation for Parkinson’s Research. H.R.M. is supported by research grants from 10.13039/501100000304Parkinson's UK, the Cure Parkinson’s Trust, the 10.13039/100011707PSP Association, the 10.13039/501100000265Medical Research Council, and The Michael J. Fox Foundation for Parkinson’s Research. N.O. receives research grants from The Michael J. Fox Foundation for Parkinson’s Research and the United Kingdom National Institute for Health and Care research (NIHR). I.F.M. receives funding from the 10.13039/100000002National Institutes of Health (1R01NS112499, R01NS132437, and U01AG076482), the Veterans Affairs Healthcare System (I01BX005978-01A1), the 10.13039/100006309American Parkinson Disease Association (APDA), the 10.13039/100013301Parkinson's Foundation (PF), The Michael J. Fox Foundation (MJFF), and ASAP-GP2.

## Declaration of interests

A.B.S. is a co-applicant on a patent application related to C9orf72, “Method for diagnosing a neurodegenerative disease” (PCT/GB2012/052140). A.B.S. holds a contract with the CAS and is the founder of Singleton Bioscience LLC. H.R.M. is a consult for Aprinoia and AI Therapeutics H.R.M. is a co-applicant on a patent application related to C9orf72, “Method for diagnosing a neurodegenerative disease” (PCT/GB2012/052140).

## Web resources

OMIM, https://www.omim.org

## Supplemental information

Data S1. GP2 banner author list
